# Autoinflation Leading to Failure of Two-Piece Ambicor Implantable Penile Prosthesis: An Outcome from a Methodical Treatment of Recalcitrant Stuttering Priapism

**DOI:** 10.1155/2014/529037

**Published:** 2014-04-22

**Authors:** R. Charles Welliver, Adam N. Fonseca, Bradford L. West, Kevin T. McVary, Tobias S. Köhler

**Affiliations:** ^1^Division of Urology, Southern Illinois University School of Medicine, Springfield, IL 62794, USA; ^2^Louisiana State University Health Sciences Center, New Orleans, LA 70112, USA; ^3^Department of Nephrology, Springfield Clinic, Springfield, IL 62794, USA

## Abstract

*Introduction*. We present the case of a patient who received a two-piece Ambicor penile prosthesis for idiopathic recurrent “stuttering” priapism refractory to other treatment options. The patient returned unable to deflate the device due to an interesting anatomically induced mechanical failure. *Aims*. To describe the method and findings of this inflatable prosthesis failure. *Results*. Prosthesis failure occurred due to restrictive corporal diameter and the unique characteristics of fluid reservoir location in the two-piece inflatable prosthesis. The patient was successfully converted to a semirigid prosthesis with resolution of the pain that was due to his prosthesis autoinflation. *Conclusion*. Stuttering priapism remains a challenging clinical problem. Penile implantation is a reasonable long-term solution in a patient refractory to less invasive options. In patients with fibrotic corpora, a malleable device should be considered (at least temporarily) if unable to dilate comfortably to 13 mm.

## 1. Introduction

In this report, we present the case of a patient who received a two-piece Ambicor penile prosthesis for idiopathic recurrent “stuttering” priapism refractory to other treatment options. The patient returned unable to deflate the device due to an interesting anatomically induced mechanical failure that led to autoinflation.

## 2. Case

This case involves a 46-year-old man with a 13-year history of stuttering priapism. Treatment for this condition required at least 100 trips to the urologist or emergency room with multiple corporal irrigations and shunting procedures. During this 13-year period, the longest length of time he went without an episode of priapism was 6 months and frequently he would require 3-4 treatments during a single week.

His medical history is notable for hypertension, percutaneous coronary stent, and type 1 diabetes from which he developed end-stage renal disease. He later was deemed a suitable candidate for, and successfully received, simultaneous kidney and pancreas transplantation in 2011 with subsequent immunosuppression including tacrolimus, azathioprine, and prednisone.

After transplantation, he continued to have episodes of recurrent priapism treated with corporal aspiration and irrigation. He presented to our care a year after transplantation with an episode of priapism and we performed a distal T-shunt with bilateral tunneling via corporal snake maneuver successfully relieving the priapism. Knowing that he had previously failed daily phosphodiesterase type 5 inhibitors (PDE5i) as a treatment for his stuttering priapism, he was started on ketoconazole with prednisone in the postoperative period. He was originally dosed at 400 mg (200 mg BID) of ketoconazole with a complementary 20 mg (5 mg QID) dose of prednisone. Routine serum testosterone levels were monitored to appropriately titrate the ketoconazole dosage and the patient's tacrolimus dose was also adjusted. At the initiation of ketoconazole, his serum creatinine was 1.8 mg/dL. After 30 days of treatment, his total testosterone was measured at 103 ng/dL and ketoconazole was decreased to 200 mg daily. At this dose he noted typical signs of low testosterone (decreased libido and energy) but continued to have functional erections when desired. After several months without an episode of priapism, he experienced another episode and ketoconazole was increased to 300 mg daily.

One week after increasing his ketoconazole to 300 mg, his creatinine escalated to 2.8 mg/dL. A transplant renal biopsy was performed, showing histologic findings consistent with a thrombotic microangiopathy (TMA). The negative C4d immunohistochemical stain and negative donor specific antibody excluded humoral rejection as an etiology of the TMA. In addition, PCR for hepatitis B and C, polyoma, herpes simplex, coxsackie, parvo, and Epstein-Barr virus studies were all negative. The tacrolimus was suspected to be the primary cause of TMA, and he was switched to sirolimus. A later repeat renal biopsy exhibited continued allograft dysfunction and the pathology demonstrated persistent TMA. The ketoconazole was now also considered a possible cause of his graft dysfunction and was discontinued as a precaution.

Without ketoconazole, the patient then opted for penile prosthesis insertion to definitively treat his stuttering priapism. Given his surgical history and the possibility of future abdominal surgery, a two-piece Ambicor inflatable penile prosthesis (American Medical Systems, Minnetonka, MN) was selected to avoid potential future intra-abdominal reservoir complications. During surgery, the corporal space was dilated with a 12 mm Brooks dilator proximally and distally and the corpora were measured to be a total of 15 cm bilaterally. The corporal tissue and tunica albuginea were noted to be abnormally stiff and difficult to dilate. A two-piece Ambicor prosthesis that was 14 cm long × 12.5 mm wide with 1 cm rear-tip extenders was implanted with difficulty but no complications. When the device was inflated, there was good symmetric inflation and the device cycled completely. The penis was straight and the cylinders were seated appropriately.

The patient returned to clinic one week later as he was unable to deflate the implant and he was experiencing significant discomfort. Attempts to manually deflate the device in the office were unsuccessful. After multiple attempts in the office, the patient could no longer tolerate attempts at deflation and he was scheduled to go to the operating room to attempt manual deflation during an exam under anesthesia with the possibility of device exploration/revision.

During the exam under anesthesia in the operating room, more forcible attempts were able to partially and temporarily deflate the prosthesis, but the cylinders were then noted to reinflate. Decision was made to surgically explore the prosthesis and investigate the restrictive process causing the auto-inflation. General anesthesia was induced and the patient underwent a standard skin preparation and draping.

We dissected into the scrotum via a penoscrotal approach and freed the pump apparatus; there were no signs of infection or hematoma. The pump was brought up into the wound and we cycled the prosthesis. Even with the pump dissected free, the prosthesis would only temporarily deflate before reinflation was noted. The previous corporotomies were opened and the prosthesis could still only be deflated temporarily. We suspect that the prosthesis was failing deflation due to the restrictive diameter of the scarred proximal corpora placing continued pressure on the fluid reservoirs in the base of the cylinders. Decision was made to remove the device and it cycled appropriately once explanted.

A modified Mulcahy washout was then performed. Using Brooks dilators the corporal diameter was calibrated at a very snug 12 mm; the corpora would not accept the 13 mm dilator. Corporal length was measured at 16 cm, which was a centimeter longer than before the prosthesis.

We decided to use a malleable implant to avoid future problems with auto-inflation in this patient with scarred, restrictive corpora. A Spectra semirigid penile prosthesis (American Medical Systems, Minnetonka, MN) that was 12 cm in length with 3 cm rear-tip extenders was selected. We intentionally used an undersized malleable prosthesis to reduce the risk of future distal erosion given his previous snake procedure. He has subsequently been followed up in clinic and is now without complaints of pain.

## 3. Discussion

Priapism is defined as a prolonged, painful penile erection lasting >4 h in the absence of sexual arousal [1]. Low-flow (ischemic) priapism involves a lack of arterial blood flow into the penis which, if untreated, can lead to progressive ischemia of the smooth muscle with corporal necrosis and subsequent fibrosis and erectile dysfunction [2].

Stuttering priapism is a subset of ischemic priapism often seen in patients with sickle-cell disease. The condition consists of frequent and recurrent episodes of priapism with clinical presentation similar to ischemic priapism but often self-limited and lasting <3 h [3]. Some patients, like the one presented in this case, do not have sickle-cell disease yet present with recurrent painful episodes of priapism with no obvious underlying etiology. These patients are considered to have idiopathic stuttering priapism.

Treatment options for stuttering priapism range from conservative pharmacotherapy to surgical intervention. Although by definition stuttering priapism is self-limited, patients with stuttering priapism can progress to prolonged (>4 hours) ischemic priapism requiring immediate intervention such as corporal aspiration with or without irrigation and *α*-receptor agonist injection into the corpora cavernosum [1].

Prophylactic treatment in patients with recurrent priapism may include using hormonal manipulation to suppress circulating free testosterone. This can be accomplished with gonadotropin releasing hormone agonists or antagonists to downregulate the pituitary gland, diethylstilbestrol to inhibit pituitary feedback, antiandrogen blockade of the androgen receptor or ketoconazole to reduce adrenal and testicular testosterone production. Other options utilizing different mechanisms include oral medications such as pseudoephedrine, digoxin, terbutaline, etilefrine, PDE5i, and gabapentin [3]. For nocturnal stuttering priapism, we have had success utilizing low dose diazepam [4]. In adequately trained patients, acute episodes can be managed early at home with self-injection into the corpora cavernosum of a sympathomimetic agent such as phenylephrine [5]. Should these more conservative measures fail, a shunt is surgically created between the corpus cavernosa and the glans or corpus spongiosum [3].

In the event that neither conservative treatment nor shunting adequately manage the patient's recurrent priapism, a penile prosthesis may be implanted to allow return to sexual activity and preserve penile length [6]. This procedure can be done in the setting of acute priapism, relieving acute symptoms and preventing subsequent erectile dysfunction, corporal fibrosis, and penile shortening. Although it is reasonable to implant a 3-piece inflatable prosthesis in the acute setting, some authors prefer a malleable device as an initial (or interim) prosthesis. The patient may then later elect an additional procedure to implant an inflatable device [2, 6].

In the case presented, the patient is an organ transplant recipient with a long history of stuttering priapism. These acute events were managed with an armamentarium of options including PDE5i, phenylephrine irrigation, and shunting. Ultimately, while ketoconazole adequately controlled his stuttering priapism, his worsening renal function led us to discontinue the medication.

It has been reported that if stuttering priapism is successfully managed for a period of time (weeks to months), the condition will not recur and the patient can eventually be withdrawn from treatment [3]. Unfortunately, this did not occur with this patient and as conservative options were now exhausted, he elected for penile prosthesis.

The Ambicor cycles fluid from the reservoir (proximal corporal cylinders) into the distal portion of the cylinders to achieve erection. Likely these recurrent ischemic bouts caused our patient to have scarred, unhealthy corpora. As the fluid reservoir for the Ambicor is the proximal portion of the cylinders, the continued pressure on this area led to autoinflation of the device as the fluid was forced forward into the distal cylinders. While the Ambicor has a mechanical failure rate as low as 0.7 to 2.3% in previous studies [7, 8] the condition seen in this patient is a previously unreported complication and failure. One previous report describes a failure to deflate, although this was due to a blood clot near the corporal input tubing [9].

## 4. Conclusion

Stuttering priapism remains a challenging patient condition. Penile implantation is a reasonable long-term solution in a patient refractory to less invasive options. In patients with fibrotic corpora, a malleable device should be considered (at least temporarily) if unable to dilate comfortably to 13 mm.

## References

[1] Montague DK, Jarow J, Broderick GA (2003). American urological association guideline on the management of priapism. *Journal of Urology*.

[2] Rees RW, Kalsi J, Minhas S, Peters J, Kell P, Ralph DJ (2002). The management of low-flow priapism with the immediate insertion of a penile prosthesis. *BJU International*.

[3] Kheirandish P, Chinegwundoh F, Kulkarni S (2011). Treating stuttering priapism. *BJU International*.

[4] Escudero RM, Fernandez ER, Garcia EL, Jimenez JT, Alonso AH, Fernandez. CH (2010). Stuttering priapism: case report and bibliographic review. *Archivos Espanoles de Urologia*.

[5] Steinberg J, Eyre RC (1995). Management of recurrent priapism with epinephrine self-injection and gonadotropin-releasing hormone analogue. *Journal of Urology*.

[6] Ralph DJ, Garaffa G, Muneer A (2009). The immediate insertion of a penile prosthesis for acute ischaemic priapism. *European Urology*.

[7] Lux M, Reyes-Vallejo L, Morgentaler A, Levine LA (2007). Outcomes and satisfaction rates for the redesigned 2-piece penile prosthesis. *Journal of Urology*.

[8] Levine LA, Estrada CR, Morgentaler A (2001). Mechanical reliability and safety of, and patient satisfaction with the ambicor inflatable penile prosthesis: results of a 2 center study. *Journal of Urology*.

[9] Murphy AM, O’Leary M (2005). Failure of the ambicor inflatable penile prosthesis to deflate. *International Journal of Impotence Research*.

